# On the nuclear pore complex and its emerging role in cellular mechanotransduction

**DOI:** 10.1063/5.0080480

**Published:** 2022-03-10

**Authors:** Atsushi Matsuda, Mohammad R. K. Mofrad

**Affiliations:** 1 Molecular Cell Biomechanics Laboratory, Departments of Bioengineering and Mechanical Engineering, University of California Berkeley, Berkeley, California 94720, USA; 2 Molecular Biophysics and Integrative Bioimaging Division, Lawrence Berkeley National Laboratory, Berkeley, California 94720, USA

## Abstract

The nuclear pore complex (NPC) is a large protein assembly that perforates the nuclear envelope and provides a sole gateway for traffic between the cytoplasm and the nucleus. The NPC controls the nucleocytoplasmic transport by selectively allowing cargoes such as proteins and mRNA to pass through its central channel, thereby playing a vital role in protecting the nuclear component and regulating gene expression and protein synthesis. The selective transport through the NPC originates from its exquisite molecular structure featuring a large scaffold and the intrinsically disordered central channel domain, but the exact mechanism underlying the selective transport remains elusive and is the subject of various, often conflicting, hypotheses. Moreover, recent studies have suggested a new role for the NPC as a mechanosensor, where the NPC changes its channel diameter depending on the nuclear envelope tension, altering the molecular transportability through this nanopore. In this mini-review, we summarize the current understandings of the selective nature of the NPC and discuss its emerging role in cellular mechanotransduction.

## I. INTRODUCTION

**FIG. 1.**
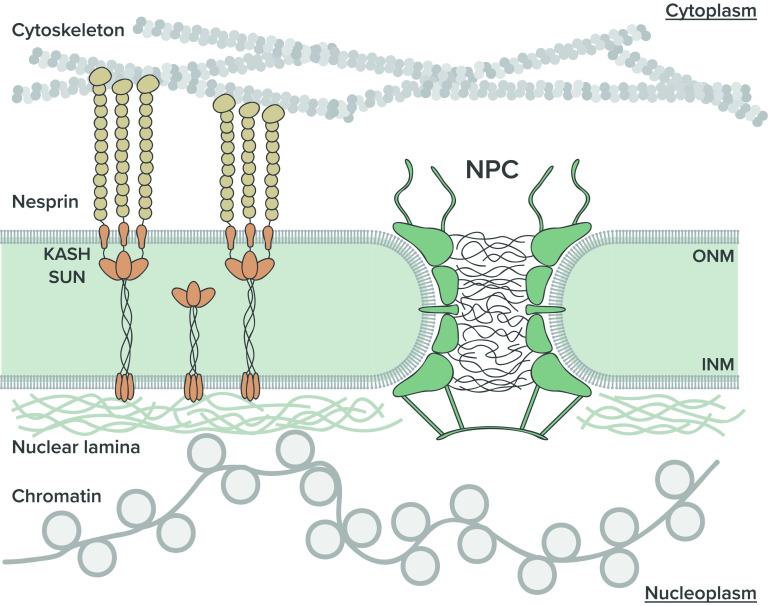
Schematics of the nuclear envelope and transmembrane proteins. The LINC complex, made up with SUN and KASH domains, connects the nuclear components, such as nuclear lamina and chromatins, to the cytoskeletons, such as actin, intermediate filament, and microtubule. The NPC perforates the nuclear envelope providing a molecular pathway between the nucleus and the cytoplasm. ONM: outer nuclear membrane; INM: inner nuclear membrane.

The genetic information of eukaryotic cells is packaged within a double-layered nuclear envelope (NE), which comprises an inner and outer nuclear membrane (INM and ONM). Several transmembrane proteins are located in the nuclear envelope. Chief among them are two integral protein complexes that span the nuclear envelope and connect the inside of the nucleus to the cytoplasm, namely, (i) the nuclear pore complex (NPC) that tunnels the nuclear envelope and acts as an exclusive gateway for molecular traffic into and out of the nucleus and (ii) the LINC (linker of the nucleus and cytoskeleton) complex that physically bridges the nucleoskeleton and the cytoskeleton (Fig. 1). By providing chemical and physical linkages across the nuclear envelope, respectively, the NPC and the LINC complex are believed to play important roles in cell and nuclear mechanotransduction.^1^ Recent studies suggest that mechanotransduction on stiff substrates is dominated by focal adhesions that are directly linked to the nucleus.^2–7^ Although the molecular mechanisms of mechanosensing and force transmission across the cell plasma membrane at the sites of cell adhesion have been extensively studied, the mechanisms of mechanotransduction at the nuclear envelope (NE) have remained largely elusive.

The LINC complexes provide a direct physical connection between the interior of the nucleus and the cytoplasm.^8,9^ The tethering of the extracellular matrix (ECM), the cytoskeleton, and the nucleoskeleton mediated by these complexes allow for a direct transmission of forces to the nucleus.^1,10–13^ Transmission of forces through LINC complexes has been shown essential for several basic biological functions of the cell including polarization, differentiation, division, and migration and other processes dependent on nuclear deformation and positioning. LINC complexes are composed of SUN (Sad-1 and Unc) and KASH (Klarsicht, ANC-1, Syne Homology) domains containing proteins, which interact in the perinuclear space (PNS) (Fig. 1). Recent studies have shown that the successful transmission of forces from the cytoskeleton to nucleoskeleton relies on a strong interaction between the SUN domains of SUN-domain containing proteins with a small peptide of KASH domain proteins in the nuclear envelope.^14,15^ Insights into molecular mechanistic regulatory roles of LINC complex proteins in sensing and responding to mechanical stimuli can allow a significant development in the understanding of cellular mechanotransduction and the role of these proteins in health and disease (see recent review^16^).

While the LINC complex is the physical connector of the cytoskeleton to the nucleus, exquisite nanochannels called nuclear pore complex (NPC) offer the sole passageway for bidirectional transport of vital cargos, ranging from different functional proteins to RNAs and ribosomes, between the cytoplasm and the nucleus in eukaryotic cells.^17^ The complex, yet delicate, geometry of the NPC and the fine spatiotemporal resolution at which the nucleocytoplasmic transport takes place have so far hindered the direct, experimental investigation of this nanomachinery. Using a hybrid of state-of-the-art experimental techniques and computational modeling approaches, ranging from continuum mechanics and coarse-grained Brownian dynamics to molecular dynamics and new agent-based modeling methods to statistical thermodynamics and bioinformatic approaches, researchers have conducted a multifaceted inquiry into the structure and function of the nuclear pore complex and the dynamics of nucleocytoplasmic traffic.^18–43^ Understanding the biomechanics of the nuclear pore complex and nucleocytoplasmic transport is anticipated to broadly impact our understanding of viral diseases and will ultimately revolutionize therapeutic approaches (e.g., gene therapy) and will also open the door to many industrial applications of biomimetic artificial nanopores.^17,27,44–46^

In this minireview, we focus on the NPC and discuss recent discoveries in relation to nucleocytoplasmic transport. We first review the structure (Sec. II) and function (Sec. III) of the NPC in relation to some recent findings. Then we discuss the suggested contribution of the NPC to the mechanotransduction (Sec. IV). We highlight the importance of this nanopore in the regulation of cellular mechanotransduction.

## II. THE STRUCTURE OF THE NPC

**FIG. 2.**
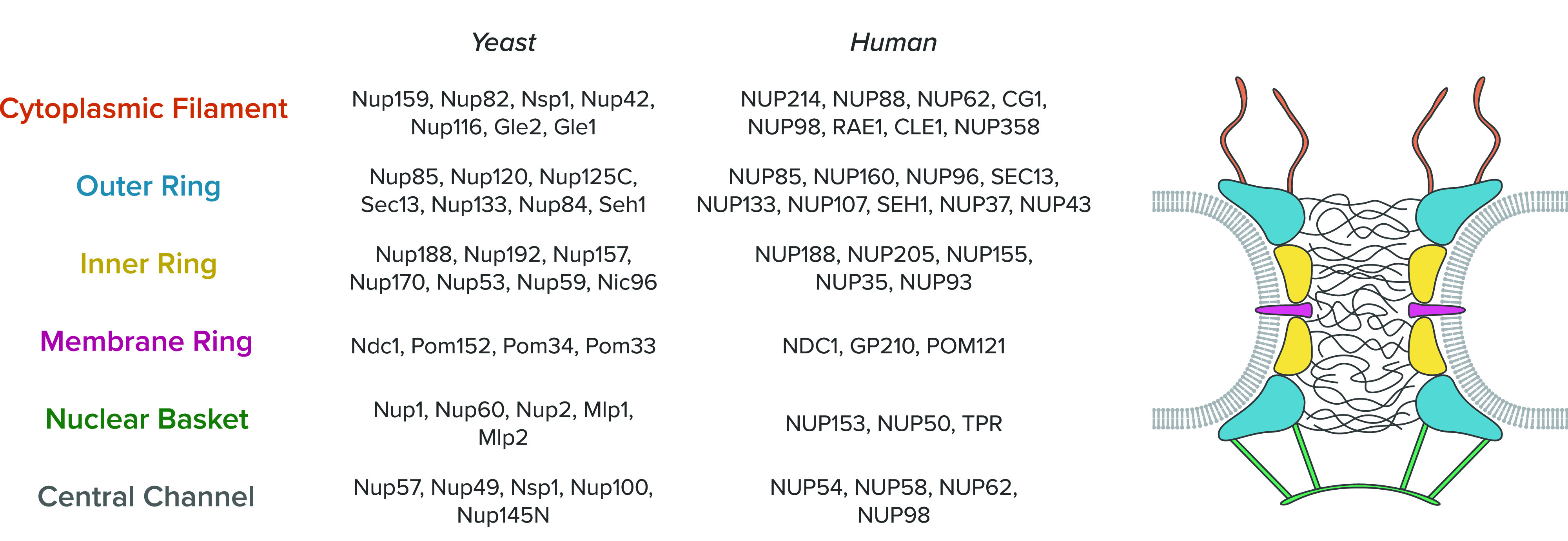
Molecular compositions of the NPC. The NPC is made up of four building blocks, namely, cytoplasmic filament, structural scaffold (outer ring, inner ring, and membrane ring), nuclear basket, and central channel. Nups included in each sub-complex are listed for yeast and human NPC. The right panel shows the overall architecture and the location of each sub-complex within the NPC.

The NPC is a large (65–120 MDa) protein assembly embedded in a nuclear envelope (NE).^17,42^ The NPC is composed of some 30 different proteins, generally referred to as nucleoporins (Nups).^18^ Each Nup is presented in multiples of eight copies, and the estimation of the total number of Nups per NPC is 500–1000,^28,37^ which varies depending on the species. The Nups collectively create sub-complexes in the NPC, classified into four groups: the structural scaffold, the central channel, the cytoplasmic filaments, and the nuclear basket^47–49^ (Fig. 2). The structural scaffold (containing ∼1/2 of all Nups) builds a platform to shape the whole structure of the NPC, anchoring the NPC to the NE. The central channel (containing ∼1/3 of all Nups) is the main nanopore domain where molecules diffuse through for the nucleo-cytoplasmic transport; the central channel is filled with the intrinsically disordered proteins called phenylalanine- and glycine-rich Nups (FG-Nups). The cytoplasmic filament and the nuclear basket protrude from the NPC into the cytoplasmic and nucleoplasmic sides, respectively. It is postulated that they may function as the docking sites for some molecules initiating nucleo-cytoplasmic transport,^1,39,50,51^ but their functional role is still under debate. In Subsections II A and II B, we review some molecular details about the structural scaffold and the central channel of the NPC.

### A. Structural scaffold

The structural scaffold is composed of eight identical sub-units called spokes, which are arranged symmetrically around the central axis of the NPC.^52–54^ The eightfold rotational symmetry maximizes the bending stiffness of each spoke and stabilizes the whole scaffold against structural distortions.^26^ The spokes are radially connected to form the concentric sub-complexes, namely, inner ring, outer ring, and membrane ring. Two inner rings run parallel along the equatorial plane of the NPC, which are sandwiched with two outer rings. The outer rings are named cytoplasmic ring and nucleoplasmic ring, depending on whether it is on the cytoplasmic or the nuclear side. The membrane ring penetrates the perinuclear lumen and harbors the whole scaffold to the NE. All of these ring complexes are joined to each other by an extensive network of short linear motifs (SLiMs),^55,56^ which exist in the intrinsically disordered regions of some Nups. The relatively weak but multiple interactions via SLiMs give flexibility as well as integrity to the structural scaffold.^57,58^

The inner ring is the most conserved module in the structural scaffold; its overall morphology and dimension are similar among different species.^52,59–62^ The diameter of the inner ring changes depending on the energy state from ∼40 nm for the constricted state to ∼60 nm for the dilated state.^63^ The inner ring radially spans from the NE to the central channel. At the periphery of the NE, the inner ring is connected to the membrane either directly or via the membrane ring. The direct bindings between the inner ring and the NE are mediated through the membrane-binding motifs (MBMs),^64–66^ which are amphipathic short amino acid sequences inserted into the membrane lipid bilayer. In the face of the central channel, the inner ring serves as the anchoring point for FG-Nups. The structured domains of FG-Nups, i.e., coiled-coil and *β*-sheet motifs, are considered to provide the grafting link to the inner ring,^23,67^ but the exact anchoring spots for them remain elusive.^68^ Most of the inner ring components are formed by either *α*-helical solenoid or a combination of N-terminal *β*-propeller and C-terminal *α*-helical solenoid,^69^ which provide flexibility and elasticity to the structure.^70^

The outer ring shows the significantly diverse size and conformation from species to species^47^ with an outer diameter of ∼98 nm for yeast and ∼120 nm for humans.^54,61^ The outer ring exists both on the nuclear and the cytoplasmic sides, harboring the nuclear basket and the cytoplasmic filaments, respectively. The outer ring has a connection to the NE via MBMs,^71^ through which they regulate the NE curvature at the inner and outer membrane fusion.^72^ Despite its variation of the overall morphology, the outer rings share the conserved building block so-called Y-complex.^73^ Y-complex comprises six to nine Nups containing *α*-helical solenoid and *β*-propeller,^74^ and it has a characteristic Y-shape. Y-complexes are arranged in a head-to-tail fashion to form the ring structure, and the set of eight complexes builds one ring.^75^ While the yeast NPC contains one ring on each side, i.e., one ring per nucleoplasmic or cytoplasmic ring, the human NPC contains two rings arranged in parallel on top of each other.^47^ As a result, there are 16 copies of Y-complexes in the yeast NPC and 32 copies in the human NPC. As one possible explanation, the difference in the number of Y-complexes stems from the different thickness of the NE (∼25 nm for yeast and ∼40 nm for humans).^62,76,77^ Still, the exact cause for the outer ring's structural variation remains unknown.

### B. Central channel and FG-Nups

The central channel is a 40–60 nm in diameter conduit surrounded by the structural scaffold,^52,63^ through which molecules diffuse for the nucleo-cytoplasmic transport. The central channel is filled with intrinsically disordered proteins called FG-Nups,^18^ whose one end is tethered to the structural scaffold while the other end dangles freely inside the channel. There are 200–300 FG-Nups in the NPC,^42^ collectively forming a molecular “cloud,” i.e., relatively high-density region of them, so-called transporter.^78^ The transporter is a dynamical entity that changes its overall shape and the internal density map over time due to the unfolded nature of FG-Nups.^21,32,33^ The transporter has several stable configuration states, namely, the “open” and “closed” states, where FG-Nups assemble near the channel wall or around the central channel axis, respectively.^79,80^ The transporter switches between these configurational states through thermal fluctuations and selectively allows molecules to pass through the central channel. (Further details on the selective transport are provided below in Sec. III.)

There are some 10–15 subtypes of FG-Nups whose fully stretched length varies between ∼50 and 300 nm.^81^ Each subtype differs as to its net charge and the Stokes radius, yielding various shapes continually ranging from collapsed to extended structure,^81^ but their overall hydrophobicity and the unfolded nature are conserved.^23^ FG-Nups contain multiple short motifs of phenylalanine and glycine residues (FG-motifs), which mostly appear in the form of FGFG, FxFG, and GLFG.^23^ FG-motifs exist separately in the sequence by having spacer regions between the neighboring motifs.^81^ The spacer regions contain hydrophilic amino acids, which promote the unfolding of the structure.^21^ Although FG-motifs themselves are hydrophobic, the existence of the spacer regions prevents them from clustering and helps maintain the disorderedness of the structure. As a result, FG-Nups behave as highly flexible polymers with a persistence length approximately ∼0.43 nm,^22^ which is close to the backbone length per one amino acid. FG-Nups are considered to be one of the most flexible polymers among intrinsically disordered proteins,^82^ and flexibility plays a key role when regulating selective molecular transport.

Inside the central channel, FG-motifs are weakly attracted to each other via hydrophobic interactions. Since the FG–FG interaction is weak enough, FG-motifs do not make a stable connection in the central channel.^22,25,83,84^ Instead, they repetitively bind on and off to each other, giving morphological flexibility to the transporter.^85^ Interestingly, the attractive interactions between FG-motifs are perfectly balanced with the repulsive interactions of the excluded-volume effect,^86^ so on average, FG-Nups behave as ideal polymers, i.e., their morphology and dynamics are simplified with the assumption of no inter-segment interaction. Similarly, FG-motifs can have hydrophobic interactions with nuclear transport receptors (NTRs),^42,85^ molecules that aid the nucleo-cytoplasmic transport (see Sec. III for details). There are several binding pockets on the surface of NTRs, where FG-motifs form weak and transient interactions.^24,87^ Although the individual affinity between each binding pocket and FG-motif is small [their dissociation constant is 1–10 mM (Refs. 88 and 89)], the multivalency of the interaction sites and FG-motifs increases their overall avidity into 1–10 *μ*M, making the FG–NTR interaction more stable.^85^

Aside from the hydrophobic feature of FG-motifs, the electrostatic interactions mediated by the spacer regions also come into play to create the selective barrier.^29^ The spacer regions contain positively or negatively charged residues randomly distributed in their sequences. Since the evolutionary substitution rate of the spacer regions is much higher than FG-motifs,^23^ they include no specific sequential pattern essential for the molecular transport, and the functional role of the spacer region is rather limited to keeping the disorderedness of the structure. Nevertheless, the free energy landscape during the molecular transport changes significantly by adding or removing the charged residues in the FG-Nup sequences,^29^ so we cannot neglect the effect of electrostatic interactions inside the central channel. Recent studies showed that FG-Nups contain a characteristic pattern formed by positively charged residues; toward the N terminus of FG-Nups, they have large-scale patterns of positively charged residues that appear with an interval of 40–60 amino acid residues.^90–93^ This pattern is named largest positive like-charge regions (lpLCRs), and the uniqueness of lpLCRs among other intrinsically disordered proteins and their role in molecular transport have been studied extensively.

## III. REGULATION OF THE NUCLEOCYTOPLASMIC TRANSPORT AT THE NPC

The primary function of the NPC is to selectively transport molecules across the nuclear envelope. The selective transport is based on the size of the transported molecules and their association with the nuclear transport receptors (NTRs).^17,42^ While small molecules less than 5–9 nm in diameter can pass through the NPC freely,^94–96^ large molecules cannot do that unless bound to NTRs. Although the molecular transport through the NPC happens in a short time, taking 1–10 ms for a molecule to pass through the central channel,^97–99^ the selectivity is precisely maintained so that the NPC can protect the nuclear component intact. In Subsections III A and III B, we first review the detailed process of the NTR-dependent molecular transport. Next, we discuss the physical mechanism underlying the selective barrier formation inside the central channel, which has been debated for decades.

### A. NTR-dependent molecular transport through the NPC

Molecules larger than 5–9 nm in diameter are transported through the NPC only when bound to NTRs. There are various kinds of NTRs found in the cell.^17^ Depending on their role in the nucleo-cytoplasmic molecular transport, they are named importin or exportin, which helps import or export molecules into/out of the nucleus, respectively. The NTRs contain multiple hydrophobic pockets on their surface, to which FG-motifs are attracted.^24,87^ The binding pockets are characterized by their overall hydrophobicity rather than specific sequence patterns. Hence, in principle, any molecules can potentially become NTRs by modifying their surface chemical properties.^40^ In addition, there are some other features suggested as factors characterizing NTRs, including the geometrical distribution of the hydrophobic pockets,^24,41,86,100^ the mechanical flexibility of the whole molecular structure,^101^ and the existence of the unique amphiphilic structures.^102^

The NTRs repetitively bind with FG-Nups and guide their associated molecules to pass through the central channel.^85^ The molecules are transported either from the cytoplasm to the nucleus or vice versa. What determines the directionality of the transport process is the asymmetrical distribution of RanGDP (guanosine diphosphate) and RanGTP (guanosine triphosphate) across the NE.^20,103^ The Ran proteins exist abundantly in their RanGDP form in the cytoplasm and RanGTP form in the nucleus. This asymmetry is created by the localization of RanGAP (GTPase activating protein) in the cytoplasm and RanGEF (guanine nucleotide exchange factor) in the nucleus, which catalyzes the GTP hydrolysis and GDP dehydration, respectively.^104^ Because RanGDP and RanGTP are involved in the initiation/termination of each transport cycle,^103^ the direction of molecular transport is uniquely determined, as outlined in the following.

**FIG. 3.**
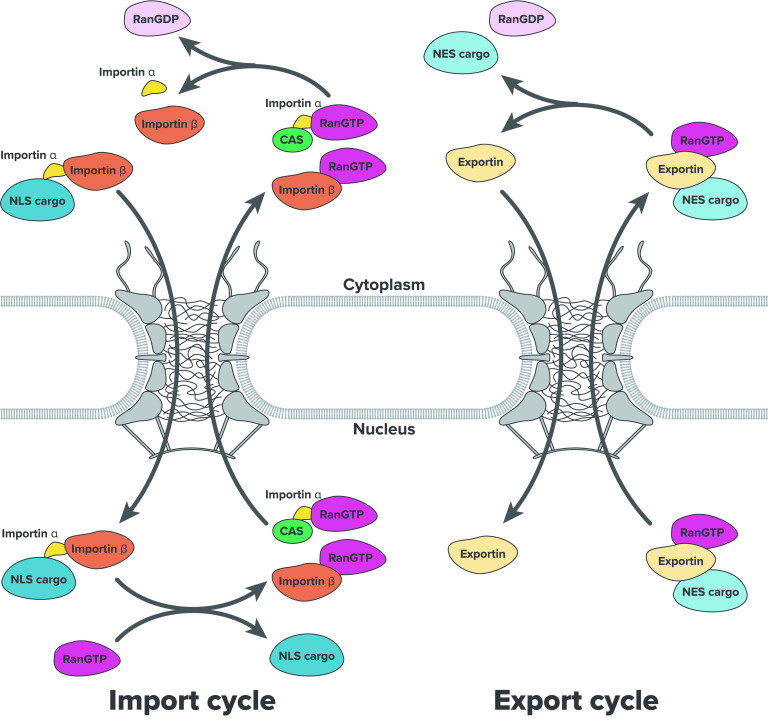
Import and export cycles through the NPC. Molecules larger than 5–9 nm in size need to be bound to NTRs (importin or exportin) to pass through NPC's central channel. Although diffusion inside the nuclear pore is a reversible process, the localized RanGTP/GDP distribution either in the nucleus or cytoplasm determines the directionality of the molecular transport.

The most-studied import pathway is the importin-dependent transport pathway (Fig. 3). This is employed by molecules containing special sequence patterns called nuclear localization signal (NLS). There are various types of NLS identified so far; classical NLS contains many arginine (R) and lysine (K) residues, and non-classical NLS has more unique compositions.^105^ Below, we explain the canonical import pathway, i.e., the most-studied pathway for NLS-containing molecules among many different pathways. In the cytoplasm, importin*α* binds to NLS and importin*β* to form a trimeric complex.^106–109^ The complex passes through the NPC using interactions between importin*β* and FG-Nups. In the nucleus, RanGTP binds to importin*β*, promoting the dissociation of importin*α* and importin*β* from the NLS-containing molecule.^103^ After the molecule is released in the nucleus, importin*β*-RanGTP complex is re-exported to the cytoplasm. Importin*α* is also exported by forming a trimetric complex with CAS (cellular apoptosis susceptibility protein) (exportin) and RanGTP.^110^ Importin*α* and importin*β* exported back to the cytoplasm are reused for the next round of the molecular import. The export pathway is similar to the import pathway (Fig. 3). In the nucleus, exportin binds to the protein containing nuclear export signal (NES) and RanGTP, forming a trimeric complex.^111,112^ The interactions between exportin and FG-Nups bring the complex to the cytoplasm, where RanGTP is hydrolyzed into RanGDP. The GTP hydrolysis results in the dissociation of RanGDP and the NES-containing molecule from exportin. After the molecule is released in the cytoplasm, the free exportin is shuttled back to the nucleus for the next cycle. It should be noted that all reactions except RanGTP hydrolysis are thermodynamically reversible.^20,113,114^ Thus, the only factor creating the directionality of the process is the asymmetric distribution of RanGTP/RanGDP. This was confirmed by the observation that reversing the RanGTP gradient between the cytoplasm and the nucleus resulted in the revered accumulation of the imported/exported molecules.^115^

### B. Physical mechanism for the selective transport

The NPC employs unique mechanisms to select molecules that can pass through it. Understanding such mysterious mechanisms has remained an active research topic for experimental, theoretical, and computational biophysicists.^42^ Unlike other transmembrane channels being responsible for the selection of ions, the NPCs transport macromolecules as large as ∼39 nm (Ref. 116) in diameter. Revealing the physical mechanism creating the selectivity would potentially open the way for a wide range of engineering applications such as nano-scale molecular filters.^27,117–120^

It is widely considered that FG-Nups in the central channel play a key role in forming the selective barrier.^121^ Depending on how to understand the dynamical state of FG–FG connections, two major models have been proposed describing the physical mechanism underlying the selective transport, namely, the virtual gate model^41,122^ and the selective phase model.^97,123^ The virtual gate model assumes that each FG–FG connection lasts only for a short period and that FG-Nups behave as non-cohesive polymers dangling freely inside the central channel.^22,25,83,84^ On the other hand, the selective phase model supposes that the FG–FG connections are stable enough to form the hydrogel of FG-Nups, which creates the mesh-like structure inside the NPC.^124,125^ In reality, FG–FG connections feature the intermediate characteristics between these two extreme assumptions, and thus, knowing both of these models is indispensable for understanding the physical mechanisms underlying NPC's selectivity. There are, of course, many other, often conflicting, hypotheses proposed to explain the selectivity at the NPC^126^ such as the polymer brush model,^127^ reduction-of-dimensionality model,^128^ and forest model.^81^

The virtual gate model^41,122^ assumes that FG-Nups are highly dynamic and constantly change their structure without being suspended by the FG–FG interactions. This assumption, together with their natively disordered nature, enables FG-Nups to form a variety of different conformations inside the central channel, thereby generating a high conformational entropy for the system. However, the conformational entropy decreases when a molecule is transported through the central channel, because it restricts the space available to FG-Nups, reducing their conformational freedom. When the size of the molecule is large enough, the reduction in conformational entropy, −ΔS, becomes non-negligible, which virtually prevents the passage of oversized molecules. On the other hand, when the transported molecule carries an NTR, it interacts with FG-motifs and changes the energy landscape of the system. Consequently, the free energy of the system changes by ΔF=ΔE−TΔS, where ΔE is the energy change associated with the NTR–FG binding and *T* is the absolute temperature. When the change in free energy, ΔF, is less than thermal energy, $k_{B}T$ ( $k_{B}$ is the Boltzmann constant), the molecule-NTR complex can pass through the NPC, which explains the size- and NTR-dependent selectivity. Several computational studies^29,36,41,118,129–133^ have shown that the effect of the conformational entropy is large enough to block passages of large cargoes. (The free energy change associated with the conformational entropy was $\Delta F=10–100\;k_{B}T$ when the size of transported molecules was larger than 5–6 nm.)^41,129^ It has been also shown that the energetic gain by the NTR–FG interactions effectively counteracts the entropic penalty, lowering ΔF to less than $k_{B}T$. Furthermore, most computational simulations yielded the dynamically moving FG-Nups inside the central channel,^33,134–137^ consistent with the virtual gate model.

The selective phase model,^97,123^ on the other hand, assumes that FG-Nups are cross-linked, forming a three-dimensional meshwork inside the central channel. The meshwork poses a diffusion barrier allowing only molecules smaller than the mesh size pass through the NPC. Meanwhile, since NTRs contain hydrophobic pockets on their surface, they can interact with FG-motifs and merge themselves into the cross-linked meshwork. By constantly rearranging the local structure of the meshwork, i.e., breaking the existing FG–FG connection and making the new FG–NTR link, NTR-carrying molecules can move through the central channel. We can view the motion of the NTR-carrying molecule as “binding-diffusion,” where the molecule switches between FG-bound and FG-non-bound states while undergoing the Fickian diffusion.^138,139^ Theoretical and computational calculations have shown that the binding-diffusion not only offers selective permeability but also enhances the diffusivity of the NTR-carrying molecules.^138–140^ *In vitro*, it is possible to make FG-Nups cross-linked to each other and generate an elastic FG-hydrogel.^124,125,141^ Such hydrogel produces a selective permeability that mimics NPC's function, lending support to the selective phase model. However, it is unclear if the hydrogel formation can happen in the physiological environment since the chemical conditions and the FG-Nups density required to create a saturated hydrogel *in vitro* are different from those in the cellular environment.^35^

## IV. EMERGING ROLE OF THE NPC IN THE CELLULAR MECHANOTRANSDUCTION

**FIG. 4.**
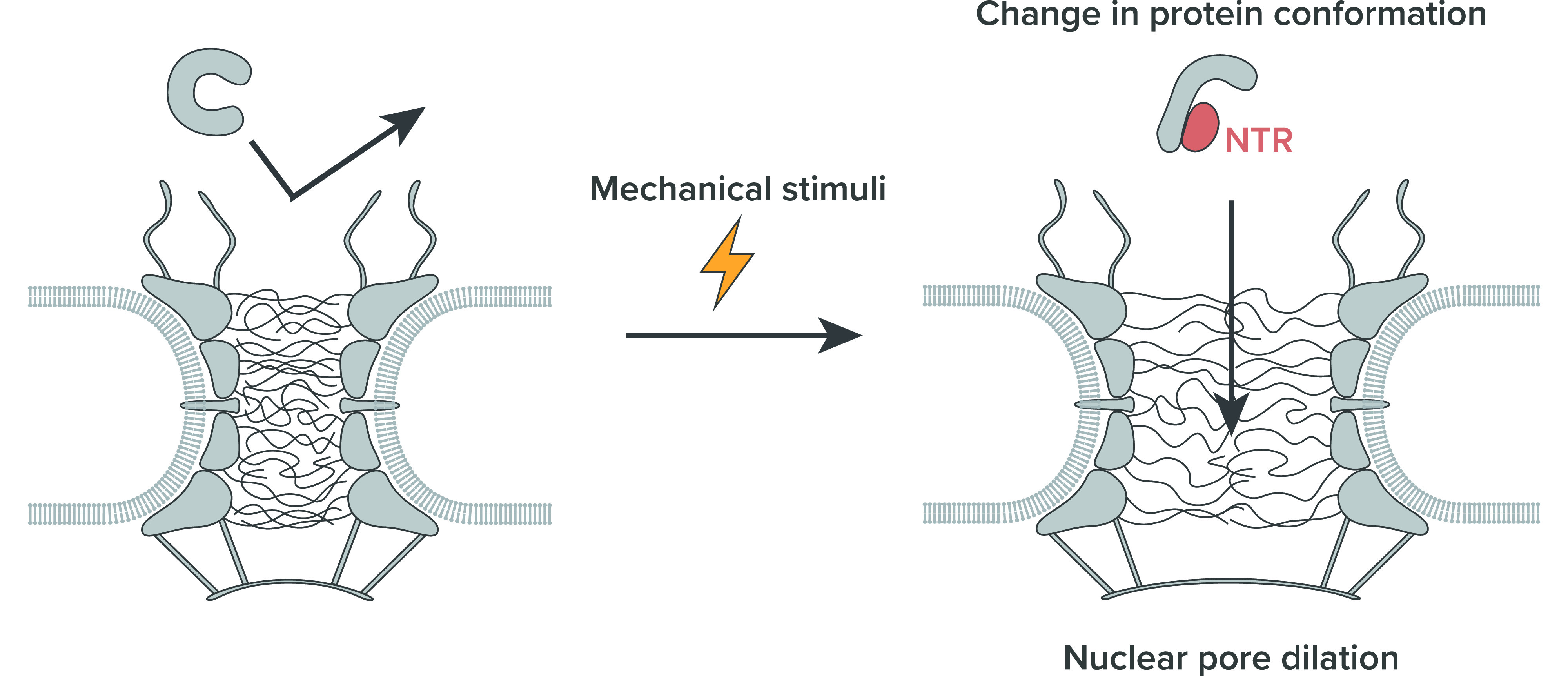
Suggested response to the mechanical stimuli. The mechanical stimuli increase the tension on the nuclear envelope, which increases the NPC diameter. The mechanical stimuli also trigger the biochemical signaling cascade, which eventually changes the conformation of the transcription factors. Both of these effects increase the transport rate of the transcription factors through the NPC.

The potential role of the NPC and nucleo-cytoplasmic transport in the regulation and mediation of mechanotransduction was originally proposed in 2009 by Wolf and Mofrad.^142^ As a molecular conduit on the nuclear envelope, the NPC aids the transport of some mechano-sensitive transcription factors into the nucleus.^142^ Upon mechanical stimuli, some transcription factors, including myocardin-related transcription factors (MRTFs), Yes-associated protein (YAP), and extracellular signal-regulated kinase (ERK), move into the nucleus, where they activate the corresponding gene transcriptions.^143^ The NPC-mediated translocation of transcription factors is a critical piece of mechanotransduction, as the dysfunction of the NPC leads to reduced mechano-sensitivity of the cell.^2,142,144^ There are two mechanisms suggested regarding how the mechanical force promotes the nuclear entry and accumulation of some specific transcription factors (Fig. 4). The first one is that the NPC increases its diameter under mechanically stretched conditions. The dilated NPC reduces the transport barrier and promotes the nuclear entry of transcription factors. The second mechanism hypothesizes that the relayed biochemical signals starting from the mechanical stimuli ultimately change the structural state of the transcription factors, changing their binding affinities with NTRs. In Subsections IV A and IV B, we further discuss these two scenarios highlighting the possible event happening at the NPC during the mechanotransduction.

### A. Nuclear pore dilation upon mechanical stimuli

The relation between the NPC pore dilation and the mechanotransduction was first suggested in 2009 by Wolf and Mofrad.^142^ The first experimental evidence for this hypothesis was presented in 2017 by Elosegui-Artola *et al.*,^152^ who demonstrated that a direct force applied to the nucleus by atomic force microscopy induces the YAP translocation into the nucleus. Because this happened without explicitly activating the biochemical signaling pathway in the cytoplasm, they suggested that increased tension on the NE membrane is sufficient to make YAP move into the nucleus. Furthermore, they speculated that the pore dilation of the NPC occurring in a high-tension NE membrane is the critical factor being responsible for the increased YAP transport rate.

Supporting the idea of the NPC pore dilation, experimentally observed conformations of the NPC display two distinct states, namely, constricted and dilated (Table I). While the constricted NPC contains a small central channel (∼40–50 nm in diameter),^52,60–63,77,132,145,149–151^ the dilated NPC has a large one (∼55–70 nm in diameter).^59,63,76,146–149^ The differences between these two states are mostly attributed to the radial contraction/expansion of the inner ring, and the overall structure of the outer ring remains nearly constant between states.^59,63^ While the constricted states are often observed in either isolated NPCs or purified NEs, the dilated states are observed in *in situ* NPCs, i.e., NPCs in the native cellular environment.^149^ This implies that the tension experienced by the nuclear envelope membrane radially stretches out the NPC causing the pore dilation.^59,148^ The transition between the constricted and dilated states in a single cell type was also observed by Zimmerli *et al.*,^63^ who demonstrated that NPCs constrict under conditions of energy depletion or hypertonic shock, both of which decrease the tension of the NE membrane.

**TABLE I.** Diameter of the inner rings for constricted and dilated NPCs. The morphologies of the NPCs were captured using cryo-(cryo-electron microscopy) and/or cryo-ET (cryo-electron tomography) in the isolated NPC sample, purified NE sample, and native cellular environment. Cryo-FIB milling technique was used to obtain NPC images in the native cellular environment except the case of Ref. 145.

	Constricted			Dilated		
	Diameter (nm)	State	References	Diameter (nm)	State	References
*H. sapiens*	41	Purified NE	60, 62, 77	66.14 ± 2.96	Native environment	146
	50	Native environment^a^	145	57	Native environment	59
	∼42.5	Purified NE	61	64	Native environment	147
*S. cerevisiae*	∼45	Isolated NPC	52	∼63	Native environment	148
	42.5	Isolated NPC	149	58.5	Native environment	149
*S. pombe*	48.6 ± 3.2	Native environment^b^	63	68.8 ± 7.9	Native environment	63
*X. laevis*	46	Purified NE	150			
	49	Purified NE	132 and 151			
*C. reinhardtii*				64	Native environment	76

^a^ Without cryo-FIB milling.

^b^ In the energy depleted cells.

One of the important factors facilitating the pore dilation is the structural flexibility of the NPC. When seen as a continuum structure, the NPC can employ a variety of different mode shapes, according to finite element models and modal analysis conducted by Wolf and Mofrad.^26^ Specifically, one of the characteristic shapes named “breathing shape” features the radially stretched scaffolds and the increased pore size, corresponding to the dilated NPC. The molecular-level details enabling such scaffold flexibility are under discussion. Solmaz *et al.* proposed that the structured domains of Nup54 and Nup58 undergo the large-scale rearrangement during the pore dilation;^153^ in the constricted state, one homotetramer of Nup58 and two homotetramers of Nup54 are stacked on top of each other building one spoke. In the dilated state, the nuclear pore complex subunit architecture reassorts into a dodecameric module to increase the pore diameter. On the other hand, the NPC architecture recently estimated by the artificial intelligence did not contain such a sub-modular level molecular rearrangement.^68^ They suggested that the individual modules in the inner ring shift inwards/outwards to change the pore diameter while preserving their modular shapes. In this model, there emerge some gaps between modules when the NPC is dilated, which are filled out by the intrinsically disordered SLiMs to maintain the integrity of the structure.^52^

Aside from the dilation of the inner ring, the structural change of the nuclear basket is also suggested as a potential response to the mechanical stimuli.^126,154^ Since the nuclear basket is extended out of the NE membrane and less restricted as to its structure, it can employ a variety of conformations without spending too much entropic cost. It is known that the nuclear basket can open or close its distal ring depending on the calcium ion concentration.^31,155^ This conformational change is driven by the electrostatic interactions between negatively charged basket arms.^156^ Additionally, direct mechanical force application can potentially work to change the nuclear basket structure.^126,154^ Since there is a direct connection between Nup153 (the nuclear basekt Nup), and SUN2 (a component of the LINC complex),^1,157^ the nuclear basket is expected to be sensitive to the external force stimuli. The combination of electrostatic and mechanical forces on the nuclear basket produces a torsional motion in the structure.^156^ A computational study by Liu *et al.*^156^ showed that in some specific parameter ranges, the conformational state of the nuclear basket has a bistable landscape, and the transition between them occurs sensitively to the mechanical force, implying its role as a mechano-sensitive molecular switch.

### B. Structural transformation of transcription factors

Another key factor that may influence the nuclear import of transcription factors is their molecular structure and their corresponding structural transformation.^158^ As discussed in Sec. III, the nucleo-cytoplasmic transport of large molecules requires their bindings with NTRs. Some transcription factors undergo conformational changes upon mechanical stimuli, expose the NLS domain on their surface and thereby increase their affinity for NTRs, promoting their passage through the NPC. On the other hand, other transcription factors do not include classical NLS in their sequences. This implies that they pass through the NPC either by using non-classical NLS or without binding to NTRs. For the latter case, the structural properties of the transcription factors play a crucial role, considering that the surface property^40^ and the mechanical flexibility^101^ are the leading factors that may modulate the transportability of molecules without NTRs. Below, we review how some transcription factors (YAP, MRTF-A, and ERK1/2) are imported into the nucleus by highlighting the possible structural changes happening during mechanotransduction. The readers may find biochemical functions of these transcription factors in the accompanying paper by Amar *et al.*^143^

MRTF-A diffuse through the NPC by binding to importin*α* and importin*β.*^159,160^ MRTF-A contains NLS in the conserved domain called the RPEL (arginine, proline, glutamic acid, and leucine) domain.^161^ Since the RPEL domain also serves as a G-actin binding site, importin and G-actin compete for binding with this domain. In the absence of mechanical stimuli, the concentration of G-actin in the cytoplasm is high enough to prevent MRTF-A from binding to importin*α*–importin*β*. On the other hand, applications of mechanical stimuli promote actin polymerization and reduce G-actin concentration in the cytoplasm. This increases MRTF-A's binding rate with importin*α*–importin*β*, and the MRTF-A–importin complex is transported into the nucleus through the NPC.

The mechanism by which YAP translocates through the NPC has remained largely unknown. Considering its relatively small size (∼65 kDa),^162^ which is slightly over the passive diffusion threshold (∼40 kDa),^17^ it might be able to adopt the passive diffusion when the nuclear pore is dilated. The possibility of the NTR-dependent diffusion is also unclear since there is no NLS (canonical or non-canonical) found within the YAP sequence. Yorkie, the *Drosophila* homolog of YAP, contains NLS which can bind with importin*α*,^163^ but it is not conserved in YAP. Future work needs to identify whether YAP passes through the NPC passively or NTR-dependently. The possibility that YAP itself can work as an NTR (by directly interacting with FG-Nups) cannot be eliminated either. Another factor to consider is the structural variation of YAP that potentially changes its transport dynamics through the NPC. YAP can undergo various post-translational modifications, including phosphorylation, O-GlcNAcylation, acetylation, and methylation.^164^ For example, when Hippo signaling is activated, large tumor suppressor (LATS) kinase phosphorylate S127. S127-phosphorylated YAP interacts with 14–3-3 protein, which leads to the sequestration of YAP in the cytoplasm. The relation between those post-translational modifications and the force application currently is under debate. Another important factor determining YAP's localization is its binding with angiomotin family proteins (AMOT).^165,166^ AMOT competitively binds to either YAP or F-actin. When the mechanical stimuli are applied, the concentration of the F-actin in the cytoplasm increases, and YAP is released from AMOT, which is followed by YAP's nuclear entry. However, there are some conflicting observations to this model, and the involvement of AMOT in the YAP's transport regulation is still unclear.^167,168^

ERK1/2, a member of the mitogen-activated protein kinase (MAPK) family, can pass through the NPC by either free diffusion,^169^ NTR-dependent diffusion,^170^ or direct interaction with FG-Nups.^171,172^ ERK1/2 does not have a canonical NLS. Instead, it contains a unique nuclear translocation signal (NTS) composed of 19 amino acids.^170^ The NTS includes the SPS (serine and proline) motif, an amino acid sequence pattern made up of serine and proline residues. When the SPS motif is phosphorylated, NTS increases the binding affinity to importin7, and therefore, the SPS motif works as a molecular switch controlling ERK1/2's nuclear transportability. When a force is applied, it activates the MAPK/ERK signaling pathway. This leads to the phosphorylation of ERK1/2, which releases ERK1/2 from its cytoplasmic sequestrators^173^ and/or promotes its interaction with importin7, leading its rapid translocation into the nucleus.^174,175^

## V. CONCLUSIONS AND OPEN QUESTIONS

In this mini-review, we discussed the basic structure and functions of the NPC along with its emerging roles in cellular mechanotransduction. By controlling the import of transcription factors into the nucleus and the export of mRNA out of the nucleus, the NPC can play a vital role in mechanotransduction. Two hypotheses have been proposed for how the NPC can accomplish this role, as follows: The first hypothesis proposes that the NPC nanopore dilates under mechanically stretched conditions, reducing the transport barrier and promoting the nuclear entry of transcription factors or the exportation of mRNA. The second hypothesis suggests that mechanotransduction signals ultimately lead to conformational change of the transcription factors, altering their binding affinities with NTRs. Both these scenarios and their potential underlying mechanisms were discussed here. As to the nuclear pore dilation hypothesis, several open questions remain to be addressed. First, the molecular arrangement of the structural scaffold in its dilated and constricted states needs to be resolved. With the current model of the NPC assembly, some gaps emerge between sub-complexes of the dilated inner ring.^59^ It is not clear if the gaps are formed by coiled-coil domains that appear by refolding the sub-complexes^153,176^ or are filled with the extensive network of SLiMs. Depending on the molecular architecture of the dilated state, the energy input needed for the rearrangement of the sub-complexes changes, which determines the mechanosensitivity of the NPC. Second, the molecular links that transmit the mechanical force to the NPC must be identified. The tensional stress that radially stretches the NPC comes from either the NE, the LINC complex, or some other connections between the cytoskeletal elements and the NPC.^1^ Identification of the force transmission pathway would provide better insight into the targeted activation or inhibition of the mechanotransduction process. Third, further investigations are needed to understand the effect of the pore dilation on the nucleo-cytoplasmic transport. While the exact mechanism for the selective molecular transport at the NPC remains unknown, there are many possibilities for how the pore dilation may influence the selectivity of the NPC. It is intuitive enough to expect the pore dilation increases the passage rate of molecules without NTRs. Still, its effect on the transportability of the NTR-carrying molecules would be more complicated and differ among different models.

## Data Availability

Data sharing is not applicable to this article as no new data were created or analyzed in this study.
